# A Large-Scale Genome-Based Survey of Acidophilic Bacteria Suggests That Genome Streamlining Is an Adaption for Life at Low pH

**DOI:** 10.3389/fmicb.2022.803241

**Published:** 2022-03-21

**Authors:** Diego Cortez, Gonzalo Neira, Carolina González, Eva Vergara, David S. Holmes

**Affiliations:** ^1^Center for Bioinformatics and Genome Biology, Centro Ciencia & Vida, Fundación Ciencia & Vida, Santiago, Chile; ^2^Facultad de Medicina y Ciencia, Universidad San Sebastian, Santiago, Chile

**Keywords:** genome reduction, genome streamlining, extremophile, acidophile, chemolithoautotroph, gene gain and loss, protein size reduction and expansion, evolution of acid resistance

## Abstract

The genome streamlining theory suggests that reduction of microbial genome size optimizes energy utilization in stressful environments. Although this hypothesis has been explored in several cases of low-nutrient (oligotrophic) and high-temperature environments, little work has been carried out on microorganisms from low-pH environments, and what has been reported is inconclusive. In this study, we performed a large-scale comparative genomics investigation of more than 260 bacterial high-quality genome sequences of acidophiles, together with genomes of their closest phylogenetic relatives that live at circum-neutral pH. A statistically supported correlation is reported between reduction of genome size and decreasing pH that we demonstrate is due to gene loss and reduced gene sizes. This trend is independent from other genome size constraints such as temperature and G + C content. Genome streamlining in the evolution of acidophilic bacteria is thus supported by our results. The analyses of predicted Clusters of Orthologous Genes (COG) categories and subcellular location predictions indicate that acidophiles have a lower representation of genes encoding extracellular proteins, signal transduction mechanisms, and proteins with unknown function but are enriched in inner membrane proteins, chaperones, basic metabolism, and core cellular functions. Contrary to other reports for genome streamlining, there was no significant change in paralog frequencies across pH. However, a detailed analysis of COG categories revealed a higher proportion of genes in acidophiles in the following categories: “replication and repair,” “amino acid transport,” and “intracellular trafficking”. This study brings increasing clarity regarding the genomic adaptations of acidophiles to life at low pH while putting elements, such as the reduction of average gene size, under the spotlight of streamlining theory.

## Introduction

Significant differences in genome sizes (number of base pairs per genome) have been detected between closely related lineages of prokaryotes isolated from a broad spectrum of environments, with genome sizes down to 1.2 Mbp in free-living bacteria (Konstantinidis and Tiedje, 2004; Dufresne et al., 2005; Lynch, 2006; Giovannoni et al., 2014; Bentkowski et al., 2015; Martínez-Cano et al., 2015; Rodríguez-Gijón et al., 2021). Small or reduced genomes, also termed streamlined genomes, have been widely observed in microorganisms adapted to live in low-nutrient niches, such as cosmopolitan marine bacterioplankton (Giovannoni et al., 2005; Schneiker et al., 2006; Swan et al., 2013; Luo et al., 2014; Sun and Blanchard, 2014; Graham and Tully, 2021), rivers (Nakai et al., 2016), slow growers in anoxic subsurfaces (Chivian et al., 2008; McMurdie et al., 2009), and in a wide range of extremophiles such as bacteria adapted to supersaturated silica (Saw et al., 2008), halophiles (López-Pérez et al., 2013; Min-Juan et al., 2016), thermophiles (Sabath et al., 2013; Saha et al., 2015; Gu et al., 2021), psychrophiles (Dsouza et al., 2014; Goordial et al., 2016), and alkaliphiles (Suzuki et al., 2014). Differences in genome size have been reported for aerobes vs. anaerobes (Nielsen et al., 2021) and for microorganisms living in warmer vs. cooler environments (Lear et al., 2017; Sauer and Wang, 2019) and in bacterial pathogens (Murray et al., 2021).

The streamlining theory proposes that genome reduction is a selective process that these organisms undergo that promotes their evolutionary fitness (reviewed in Giovannoni et al., 2014). The theory suggests that a smaller genome reduces the energy cost of replication, and by encoding fewer gene products, there is a concomitant reduction of cell size that could optimize transport and nutrient acquisition (Button, 1991; Sowell et al., 2009). Some marine microorganisms with streamlined genomes have been found to have proportionately fewer genes encoding transcriptional regulators and an overall lower abundance of mRNA transcripts per cell, potentially reducing the cost of transcription and translation (Cottrell and Kirchman, 2016). These results are congruent with the observed correlation between regulatory network complexity and genome size (Konstantinidis and Tiedje, 2004). Genome size reduction is also observed in symbiotic microorganisms (Baker et al., 2010; Gao et al., 2014), but it has been theorized that this phenomenon differs to the streamlining of free-living bacteria as the former lose genes by genetic drift due to function redundancy between the host and the symbiont, while the latter would lose them by intense selective pressure (McCutcheon and Moran, 2012; Giovannoni et al., 2014), although recent evidence has argued otherwise (Gu et al., 2021).

Any organism that grows optimally at low pH can technically be classified as an acidophile. However, because there are many neutrophiles (optimum growth ∼pH 7) that successfully grow at around pH 6 or lower, it is useful from a practical point of view to define acidophiles as those microorganisms that grow optimally below pH 5 and make a distinction between moderate acidophiles that grow optimally between pH 5 and about pH 3.0 (Foster, 2004; Dopson, 2016; Benison et al., 2021) and extreme acidophiles that grow below pH 3 (Johnson, 2007). The latter are particularly challenged for survival and growth as they face a proton concentration across their membranes of over 4 orders of magnitude (Baker-Austin and Dopson, 2007; Slonczewski et al., 2009). Acidophilic microorganisms have been identified in all three domains of life (Johnson and Hallberg, 2003), but currently more genomic information is available for prokaryotic acidophiles (Archaea and Bacteria) (Cárdenas et al., 2016; Neira et al., 2020).

Our current understanding about genome streamlining in acidophiles comes from a limited number of observations. It has been reported that the genomes of several acidophilic microorganisms, such as *Methylacidiphilum*, *Ferrovum*, *Leptospirillum* (domain Bacteria) and *Picrophilus* (domain Archaea), are smaller (2.3, 1.9, 2.3, and 1.5 Mb, respectively) compared to their closest neutrophilic phylogenetic relatives (Angelov and Liebl, 2006; Hou et al., 2008; Ullrich et al., 2016; Vergara et al., 2020). Genome reduction in acidophiles has been discussed as a mechanism to reduce energy costs to survive in extremely low-pH environments where organisms must deploy multiple energy-intensive acid resistance mechanisms to maintain a circum-neutral cytoplasmic pH (Hou et al., 2008; Ullrich et al., 2016; Zhang et al., 2017; Vergara et al., 2020) while thriving in often nutrient-scarce and heavy-metal-polluted low-pH environments (Johnson, 1998; Dopson et al., 2003; Johnson and Hallberg, 2008). Despite this progress, there remains much to be discovered about genome reduction in acidophiles. With the increased availability of genome sequences of acidophiles (Cárdenas et al., 2016; Neira et al., 2020), we aim to determine whether there is a statistically supported correlation of genome reduction with low pH and, if so, what are the elements influencing this tendency. We also analyze and comment on the differences in genetic functions between acidophiles and neutrophiles that are involved in these changes.

## Materials and Methods

### Data Procurement and Management

#### Genome Information

Genomes of 345 bacterial acidophiles together with their associated growth and taxonomic data were obtained from AciDB^1^ (Neira et al., 2020). This set of genomes was modified for the present study in two ways: (i) organisms without an identified phylum affiliation were discarded and (ii) seven new genomes and their associated metadata from acidophiles have been added since the publication of AciDB. This resulted in an initial dataset of 342 genomes of acidophiles. In addition, 339 genomes were collected from non-acidophiles (growth optima, pH 5–8). These included 222 genomes of neutrophiles (growth optima, pH 6–8) that were the closest phylogenetic relatives to the acidophiles as identified using the National Center for Biotechnology Information (NCBI) taxonomy (Schoch et al., 2020), GTDB (Chaumeil et al., 2020), and AnnoTree (Mendler et al., 2019), resulting in an equal taxonomic representation of genomes of acidophiles and their neutrophilic phylogenetic relatives (Supplementary Table 1). The genome sequences were downloaded from NCBI and the Joint Genome Institute (JGI). The genomes were filtered for quality using CheckM v1.0.12, with cutoffs for completeness at > 80% and contamination at < 5% (Parks et al., 2015). This resulted in a final data set of 597 high-quality bacterial genomes, comprising 264 genomes from acidophiles (pH < 5) and 333 genomes from non-acidophiles (pH 5–8). The genome information is provided in Supplementary Table 2.

Genome average nucleotide identity was determined using fastANI v1.3 with 4 threads (Jain et al., 2018). A cutoff of 95% average nucleotide identity was defined (Kim et al., 2014) to group identical or highly similar genomes into species clusters. The genomic characteristics, proteomic data, and associated metadata are reported as the means of each group for all plots. This reduced data bias due to over-representation of some highly sequenced species.

#### Growth pH and Temperature

Data on the optimal growth pH and temperature of a species were downloaded from AciDB (Neira et al., 2020). For new species with sequenced genomes not yet deposited in AciDB, information for optimal growth pH and temperature was extracted from the literature. When no description of these optima was available, they were defined as the midpoint of the growth range reported for the strain or closely related strain as described by Neira et al. (2020). For metagenomes, the reported environmental data were used to determine optimum pH and temperature.

### Proteome Analyses

#### Protein Annotations

The genome annotations were downloaded from NCBI^2^ or JGI.^3^ Genomes without an existing annotation were annotated with prokka v1.13.3 (Seemann, 2014). A proteome table was generated for each genome, which includes information for each predicted protein, including size, predicted subcellular localization, functional annotation with Clusters of Orthologous Genes (COGs) and Pfams, COG category, and presence of signal peptide and ortholog group. Unless stated, all software was run with default options.

#### Ortholog Groups

To define ortholog groups, reciprocal BLASTP was performed within each genome by using all the proteins in its predicted proteome as queries against a database of the same proteins. A coverage of 50%, a sequence identity of 50%, and an *e*-value of 10^–5^ were used as cutoffs (Tettelin et al., 2005; Naz et al., 2020). The protein pairs that follow these conditions were assigned to the same ortholog family if one or both were the best-scored BLASTP hit of the other. Ortholog groups will also be referred to as protein families.

#### Subcellular Localization

Subcellular locations were assigned to each predicted protein using PSORTb v3.0 (Yu et al., 2010), which predicts either cytoplasmatic, inner membrane, exported, outer membrane, periplasmic for gram-negative bacteria, or cell wall for gram-positive bacteria. An “unknown” tag is assigned to proteins whose subcellular location could not be predicted. This was complemented with signal peptide identification, which was assigned using SignalP v5.0b that predicts the presence of signal peptides for translocation across the plasmatic membrane by either the Sec/SPI (standard system), Sec/SPII (lipoprotein signal peptide system), or Tat/SPI (alternative system) translocation/signal peptidases (Almagro et al., 2019). All three positive predictions were binned together and tagged as “has signal peptide”. The proteins were sorted by both subcellular localization and signal peptide presence.

#### Pfam and Clusters of Orthologous Genes Functional Annotations

Pfams were assigned to predicted proteins using Pfam_scan v1.6 (Finn et al., 2016) under Pfam version 32.0 (El-Gebali et al., 2019), which contains a total of 17,929 different functional annotations, including protein families and clans. An *e*-value of < 10^–5^ was applied as a cutoff for Pfam predictions of protein function. The Pfam with the lowest *e*-value was assigned to each protein. COG annotations were assigned with the web tool eggNOG-mapper v5.0 (Huerta-Cepas et al., 2019) under the December 2014 version of the COG database, which contains 4,632 functional annotations (Galperin et al., 2015). The percentage of ortholog groups that have a Pfam assignment (Mistry et al., 2021) or a COG assignment (Galperin et al., 2021) was calculated for each proteome. The percentage of ortholog groups belonging to each COG category was also calculated. In addition, Pfam assignments were used for the analysis of intra-protein family size variation and to determine the percentage of proteins with an annotation.

#### Paralog Frequencies

Paralog families were defined as ortholog groups with two or more proteins from the same proteome. The percentage of proteins that belong in paralog families was calculated for each COG category in relation to the total number of proteins in the category. The same procedure was repeated for the full proteome.

### Statistical Analyses

A python script was developed to gather, filter, organize, and analyze the data from the organisms’ genomes and proteomes. Data distributions were statistically analyzed using the following methods. The scipy library (Virtanen et al., 2020) was used for linear fittings (with the “linregress” module), binomial test (with the “stats.binom_test” module), and Pearson’s linear correlation coefficient (with the “stats.pearsonr” module). A two-sided mode was used for all the tests. The *P*-value thresholds used for statistical significance were 0.05, 0.01, and 0.001. For estimation of correlation in potentially heteroscedastic distributions, generalized least squares was applied using the module “regression.linear_model.GLS” within the statsmodels library (Seabold and Perktold, 2010). For multi-testing analyses, false discovery rate was used to determine the statistical significance using the Benjamini/Hochberg procedure (Benjamini and Hochberg, 1995) with the “stats.multitest.multipletests” module also within the statsmodels library. A *q*-value of 0.05 was used for Pearson’s correlation *p*-values. The *q*-value is the upper limit of the rate of the findings (null hypothesis rejections) that is expected to be a false positive. Principal component analysis (PCA) was performed with the “decomposition. PCA” module within the sklearn library (Pedregosa et al., 2011). The number of components for dimensionality reduction was set to 2. Data was plotted using the matplotlib library (Hunter, 2007).

## Results and Discussion

### Phylogenetic Distribution and Associated Metadata of the Genomes Interrogated

**FIGURE 1:**
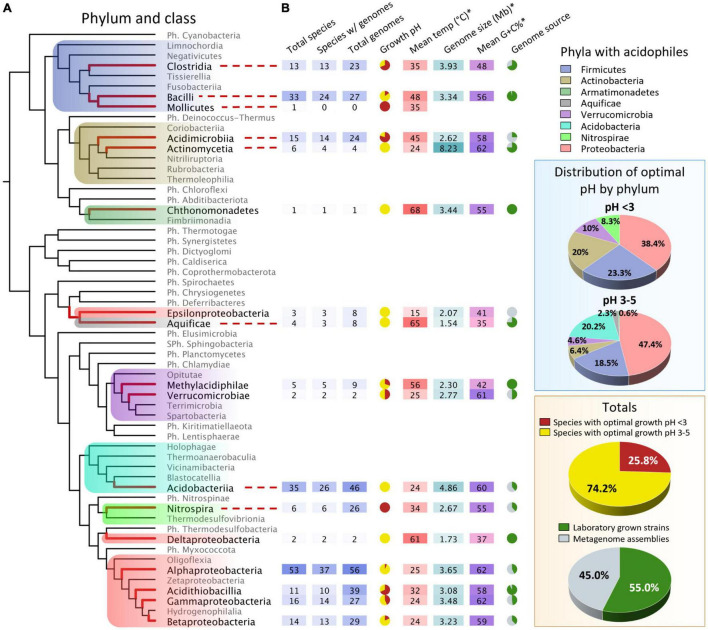
Taxonomic distribution of the acidophilic genomes interrogated. **(A)** A rooted cladogram displaying the phyla, classes, and metadata of acidophiles with genomic data. The cladogram was constructed using AnnoTree (Mendler et al., 2019) as a guide for phylogenetic positioning and rooted as described by Parks et al. (2018). The phyla with acidophiles were broken down into classes. Lineages with known acidophiles are highlighted, and their branches are shown with thick red lines. **(B)** Genomic and growth data of the taxa with acidophiles. Dashed lines connect the acidophilic lineages with the taxon’s information when necessary. Growth pH pie charts represent the percentage of species that grow optimally at pH < 3 (red) and at pH 3–5 (yellow). Genome source pie charts represent the percentage of acidophilic genomes sequenced from laboratory pure strains (dark green) vs. metagenome assemblies (gray). **(C)** Percentage of acidophilic species by phyla for both pH ranges (< 3 and 3–5). **(D)** Totals of both pie charts from **(B)** for all the phyla combined. Ph., phylum; Sph., superphylum. The asterisk indicates the mean values for the acidophiles in the taxon. A more detailed table with the classes’ information can be found in Supplementary Table 3.

From the 342 publicly available genomic sequences (264 high-quality plus 78 low-quality genomes) of acidophilic bacteria, 331 genomes with well-defined taxonomy (phylum and class) were mapped onto a rooted cladogram (Figure 1). The genome sequences come from 177 species distributed in 17 classes and 8 phyla out of a total of 37 recognized bacterial phyla (55 if candidate phyla are included) (Schoch et al., 2020; Figure 1 and Supplementary Table 3). The acidophiles are widely distributed in the cladogram, supporting the idea that acidophile lineages have emerged independently multiple times during evolution (Cárdenas et al., 2016; González et al., 2016; Colman et al., 2018; Khaleque et al., 2019; Vergara et al., 2020).

Supplementary Figure 1 shows the distribution of acidophilic species with sequenced genomes by phylum across pH, where pH represents the optimum for growth for each species. The total number of species declines from about 60 species in the range pH 4–5 to about 10 at pH 0.5–1.5, consistent with the observation that species diversity declines in low-pH environments (Bond et al., 2000; Baker and Banfield, 2003; Johnson and Hallberg, 2003; Méndez-García et al., 2014; Lukhele et al., 2020; Hedrich and Schippers, 2021). These estimates are based on the distribution of acidophiles with publicly available sequenced genomes; the true richness of acidophile diversity is likely to be much higher and will probably increase as more acidic econiches are sampled using metagenomics approaches.

**FIGURE 2:**
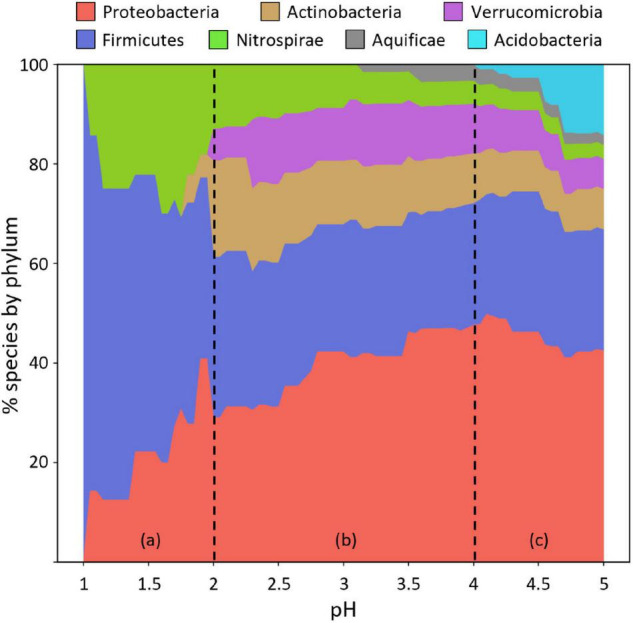
Distribution of acidophilic species with sequenced genomes by phylum across pH. Cumulative plot of relative abundance (%) of acidophiles across pH. Percentages indicate species that can live at or below a given pH. **(a–c)** Indicate pH ranges 1–2, 2–4, and 4–5, respectively. The phyla are color-coded. Phylum *Armatimonadetes* has only one acidophilic species and is not shown.

Figure 2 shows the distribution of species by percentage across pH. The results have been divided into three sections (a–c) for discussion. Section (a) with a pH range of 1.0–2.0 is dominated by species in the phyla Proteobacteria, Firmicutes, and Nitrospirae in approximately equal proportions at around pH 2 and by Firmicutes at pH 1. Section (b) shows the species distribution in the range pH 2–4. Acidophilic species of phylum Proteobacteria are the most prevalent in this range but exhibit a declining percentage with decreasing pH. Species of Actinobacteria and Verrucomicrobia are represented about equally, but both phyla have few representatives below pH 2. Species of Aquificae are present in a low percentage (∼3%), down to about pH 3, beyond which there are no representative genomes. Section (c) shows the species distribution in the range pH 4–5. All seven phyla (eight, including the one species from Armatimonadetes) have species in this range, but Acidobacteria show a declining percentage from pH 5–4, below which there are no representative genomes.

### Genome Size as a Function of pH

**FIGURE 3:**
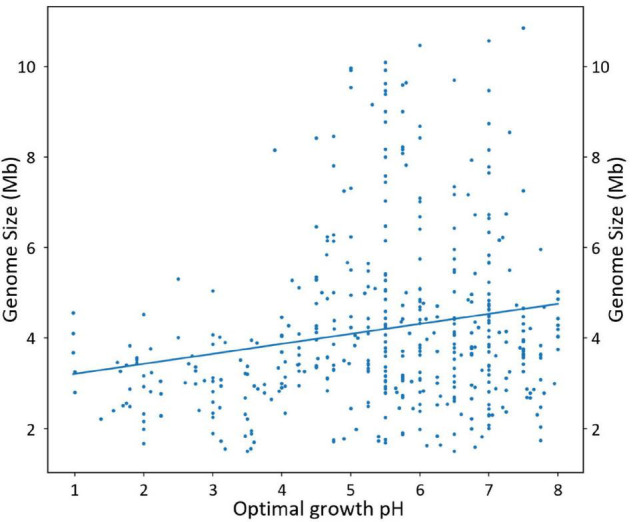
Scatterplot of the genome size (Mb) of bacterial acidophiles and their most closely related extant, circum-neutral relatives vs. optimal growth pH. Each point corresponds to a different species. A linear regression curve has been fitted to the data with a Pearson’s correlation coefficient of 0.19 and a *p*-value of 2.97 × 10^–5^. The generalized least squares *p*-value was 1.8 × 10^–3^.

A scatterplot of genome size across optimal growth pH shows declining genome sizes from about 4.5 Mb for circum-neutrophiles to an average of about 3.4 Mb for extreme acidophiles (Figure 3). There are no large genomes (> 5 Mb) for bacteria that grow below about pH 4, whereas large genomes including up to about 10 Mb are present in acidophiles that grow between pH 4 pH 5 and in neutrophilic relatives of the acidophiles that grow from pH 5 to 8. A linear regression model fitted to the data shows a tendency that is statistically significant with a positive Pearson’s correlation coefficient of 0.19 and a *p*-value of 2.97 × 10^––5^, implying that genomes are smaller at a lower pH. However, there is evidence of heteroscedasticity^4^ in the plot, which means that the variance is not constant across one of the variables (in this case, the pH), which invalidates Pearson’s correlation tests. We applied generalized least squares regression to take into account heteroscedasticity, and a *p*-value of 1.8 × 10^–3^ was obtained, supporting the proposed relationship between pH and genome size.

However, the presence of heteroscedasticity suggests the possibility that other variables, in addition to pH, may contribute to the determination of genome size. To address this issue, we investigated the potential contributions of growth temperature and genomic G + C content on the distribution of genome size across pH. Many acidophiles are also moderate or even extreme thermophiles (Johnson and Hallberg, 2003; Capece et al., 2013; Colman et al., 2018), and temperature has been suggested to be a driving force for genome reduction (Sabath et al., 2013). Genome size has also been associated with G + C content, where organisms with relatively low genomic G + C content tend to have smaller genomes (Veloso et al., 2005; Almpanis et al., 2018).

We evaluated how these factors are correlated with genome size and pH (Supplementary Figure 2). Temperature is negatively correlated with genome size (Pearson’s correlation coefficient, −0.34; *p*-value, 2.9 × 10^–13^), and G + C is positively correlated with genome size (Pearson’s correlation coefficient, 0.48; *p*-value, 1.91 × 10^–25^). A negative correlation between genome size and temperature has recently been reported for extreme acidophiles of the *Acidithiobacillus* genus (Sriaporn et al., 2021). However, no statistically supported correlation is observed between temperature and pH (Pearson’s correlation coefficient, −0.01; *p*-value, 0.84) nor between G + C content and pH (Pearson’s correlation coefficient, −0.06; *p*-value, 0.22). Therefore, while both temperature and G + C content have a strong influence on genome size, they appear to act independently of the relationship between pH and genome size.

**FIGURE 4:**
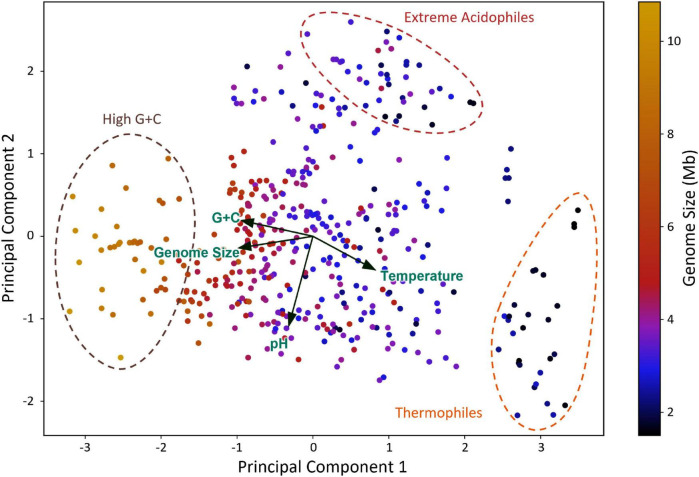
Principal component analysis (PCA) of multiple variables potentially influencing the genome size. Dimensionality reduction was performed by PCA, inputting the optimal growth pH, optimal growth temperature, G + C content, and genome size of each species in the dataset. A biplot was constructed, which shows the loadings of each variable as arrows at the center of the plot and the distribution of the principal components. The average genome size of each species is shown as a color scale. Three clusters within the dotted circles are highlighted for their distinctive features.

To investigate further the interplay of pH, temperature, and G + C content with genome size, we performed dimensionality reduction and visualization *via* PCA (Jolliffe, 2005). As seen in Figure 4, the directions of the loading vectors show that temperature is negatively correlated with both G + C content and genome size, while genome size is positively correlated with both G + C content and pH. This is also depicted in how the smallest genomes are found in thermophiles (optimal temperature: > 55°C, rightmost cluster) followed by extreme acidophiles (optimal pH: < 3, upmost cluster), while the biggest genomes are found in a high-G + C-content group (leftmost cluster). Conversely, the orthogonality of the loading vectors suggests that no correlation is observed between pH and temperature or between pH and G + C content. Therefore, when considering all variables at once, the same results are observed as when the variables were individually assessed (Supplementary Figure 2), providing additional evidence that neither G + C content nor temperature affects the correlation between pH and genome size; rather, multiple driving forces can independently exert their influence on genome size.

### Genetic Mechanisms Affecting Genome Sizing

**FIGURE 5:**
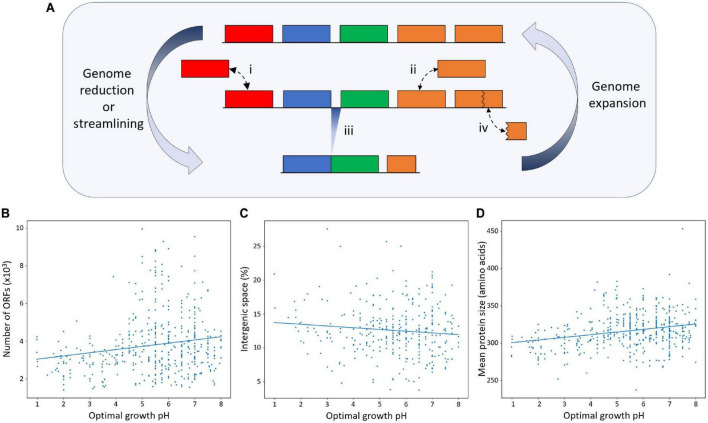
Mechanisms involved in genome size changes. **(A)** Diagrammatic representation of the genetic mechanisms involved in genome size changes. Five genes of a hypothetical genome are shown, where the top, middle, and bottom rows represent an expanded genome, a transition genome, and a streamlined genome, respectively. The orange boxes indicate paralogous genes. The processes involved in genome size changes are shown, where (i) and (ii) represent gene loss/gain of single-copy genes or paralogous genes, respectively, (iii) shows the intergenic space reduction or expansion, which we refer to as genome compaction, and (iv) shows the gene size reduction or increase. **(B)** Number of genes (ORFs, open reading frames) across pH. Pearson’s correlation coefficient is 0.18, with *p*-value 1.25 × 10^–4^. **(C)** Intergenic space vs. pH. Intergenic space is defined as the genome size minus the sum of the nucleotide length of all protein-coding genes as defined by the ORFs of a genome divided by genome size, in percentage. A stricter genome quality filter of 97% completeness and 2% contamination was used in this analysis to minimize mis-annotation errors due to fragmented genomes. In total, 394 genomes from 317 species passed the filter. Pearson’s correlation coefficient is −0.11, with *p*-value 0.06. **(D)** Average protein length across pH. Pearson’s correlation coefficient is 0.25, with *p*-value 4.03 × 10^–8^.

Given the observation that genome size is negatively correlated with pH in acidophiles, we aimed to determine what genomic processes influence this relationship. Figure 5A shows a diagrammatic representation of genetic mechanisms that have been postulated to be involved in genome expansion or reduction in Bacteria and Archaea (Keeling and Slamovits, 2005; Sabath et al., 2013; Giovannoni et al., 2014; Gillings, 2017; Kirchberger et al., 2020; Rodríguez-Gijón et al., 2021; Westoby et al., 2021). Genome size changes could result from having changes in the number of orthologous families (i, Figure 5A) or paralogous genes (ii, Figure 5A), in genome compaction/expansion resulting from changes in the number of intergenic nucleotides, including alteration in the frequency of overlapping genes (iii, Figure 5A; reviewed in Kirchberger et al., 2020), and in smaller or larger genes, including loss/gain of domains (iv, Figure 5A).

Based on the schema shown in Figure 5A, we investigated the contribution of the different mechanisms in genome size changes in acidophiles across pH. Annotated open reading frames (ORFs) were used as surrogates for “genes”. A caveat is that ORF prediction depends on the quality of the genome sequence, where poor-quality genomes frequently have incorrectly annotated chimeric and truncated ORFs that confound the subsequent identification of genes (Klassen and Currie, 2013). We minimized these potential errors by analyzing only genomes that had passed a high-quality CheckM filter (Parks et al., 2015), yielding the 597 genomes used in our genomic analyses. However, even high-quality genomes are prone to errors of ORF annotation, especially in the identification of correct translation start sites (Korandla et al., 2020), which will impact the predictions of gene and intergenic spacer sizes. Currently, there is no computational program for ORF prediction that is flawless, including GenBank (Korandla et al., 2020), and we expect that future work will improve the annotations of ORFs used in our study.

#### Reduction/Expansion of Gene Number

The number of protein coding genes (ORFs) of each genome under interrogation was plotted as a function of the optimal growth pH of the species (Figure 5B). The results indicate that there is a statistically significant reduction (Pearson’s coefficient: 0.18; *P*-value: 1.25 × 10^–4^) of the average number of ORFs per organism across pH from an average of about 4,100 ORFs/organism at pH 7 to about 3,200 ORFs/organism at pH 2 (Figure 5B). This has been regarded as possibly the most predominant mechanism for genome size changes (Konstantinidis and Tiedje, 2004), and this is likely also true for our dataset (Supplementary Figure 3).

#### Reduction of Intergenic Spacers as a Possible Contributor to Genome Compactness

It is well established that bacteria have compact genomes with an average protein-coding density of 87%, with a typical range of 85–90% (McCutcheon and Moran, 2012). Genome size reduction could occur by decreasing the amount of DNA occupied by intergenic spacers—for example, by promoting the frequency of overlapping genes (Veloso et al., 2005; Saha et al., 2015; Kreitmeier et al., 2021). This strategy has been especially exploited in compacting viral genomes (Pavesi, 2021).

To evaluate whether a reduction in the fraction of the genome dedicated to non-protein-coding DNA contributed to the smaller genomes observed in acidophiles, we calculated the percentage of intergenic spaces (IG) dedicated to the total genome content across pH. IG was calculated as genome size (Mbp)—∑Mbps of all ORFs in a genome, expressed as a percentage of the total Mbps in the genome. A smaller % IG implies greater genome compaction. A tendency was observed for % IG to increase as pH growth optima declines (Figure 5C), which is borderline statistically significant (Pearson’s coefficient = −0.11; *p*-value, 0.06). An increase in intergenic space in acidophiles is an interesting finding that might be explored further in future studies and indicates that this element is most likely not contributing to the reduced genome sizes of acidophiles. This result is particularly sensitive to the aforementioned errors of ORF annotation, and this influences the estimation of the percentage of intergenic genomic DNA.

#### Reduction/Increase of Protein Size

The average protein size was plotted as a function of pH (Figure 5D). There is a statistically supported positive correlation (*p*-value: 4.03 × 10^–8^) between average protein size and pH, with an average size of 320 amino acids at pH 7 to 300 at pH 2. This indicates that acidophiles have shorter proteins on average, which could be produced by a loss of larger proteins or by protein size reduction (Figure 5A, mechanism iv) or possibly both.

**FIGURE 6:**
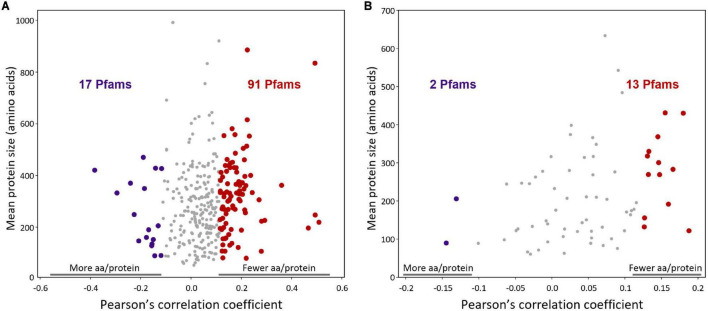
Protein size vs. pH correlations for conserved Pfams. **(A)** Pfams present in over 90% of species and in a pH span of at least 6 pH units were selected for analysis. For each Pfam, the Pearson’s correlation coefficient for protein size vs. organism optimal growth pH was calculated using the species averages as data. Each point corresponds to a different Pfam. Positive correlations (91 red points to the right) indicate Pfams whose proteins are shorter at low pH, while negative correlations (17 purple points to the left) are Pfams whose proteins are larger at low pH. The 25 Pfams with the lowest *p*-values are listed in Supplementary Table 4. **(B)** Analog to **(A)** but for a list of Pfams that in addition to being present in over 90% of the species and in a span of at least 6 pH units were also in a unique copy in the genomes (proteins with the Pfam per genome < 1.1), and only one domain architecture was dominant in the proteins. These Pfams are listed in Supplementary Table 5. For both plots, a false discovery rate *q*-value of 0.05 was used for statistical significance. Significant correlations are shown as big points, which are red for positive correlations and purple for negative correlations. Non-significant correlations are shown as small gray points.

To quantify protein size reduction in acidophiles, we analyzed the protein sizes of several conserved Pfams (> 90% of the species) in the dataset (Figure 6). We observed that the conserved Pfams with reduced protein sizes in acidophiles are over 5 times as many as the conserved Pfams with increased sizes (Figure 6A; binomial test *p*-value, 2.1 × 10^–13^). This result accounts mainly for changes in the predominant domain architectures, implying that these proteins in acidophiles likely have fewer domains—for example, the Pfam for the biotin attachment domain was mainly found without additional domains below pH 5, while in neutrophiles it can often be found next to other domains, such as dihydrolipoamide acyltransferase (Supplementary Table 4). This inclination toward protein size reduction is also observed in a collection of conserved Pfams that are also in single copy and predominantly in single-domain architectures (Figure 6B; binomial test *p*-value, 7.4 × 10^–3^). This result accounts mainly for loop size reductions and domain size reductions. Such is the case of the ribosomal protein L19 that, in acidophiles, lacks long loops and is 4 amino acids shorter on average (Supplementary Table 5). As for the possible contribution of gene gain/loss into the reduction of the average protein size in acidophiles (by gain of smaller proteins or loss of larger proteins), we estimate that it had a much less significant contribution than protein size reduction (Supplementary Figure 4).

### Gene Categories Over- and Underrepresented in Acidophiles

Having established that there is a statistically supported positive correlation between genome size and optimal pH for growth and that gene gain and loss events likely contributed to this correlation, we investigated in more detail what types of genes were involved in these events.

#### Changes in Ortholog Group Representativity in Acidophiles

To gain insight into the contribution of gains or losses of genes in the observed genome size changes of acidophiles (mechanism i, Figure 5A), we first clustered the genes into ortholog families and systematically classified the predicted proteomes of each genome by (i) subcellular location and (ii) functional category as predicted by Pfam annotations (Mistry et al., 2021) and COG categories (Galperin et al., 2015). Subsequently, we mapped the frequencies of ortholog families of these categories in the genomes across pH.

##### Changes in Ortholog Frequencies by Subcellular Location

**FIGURE 7:**
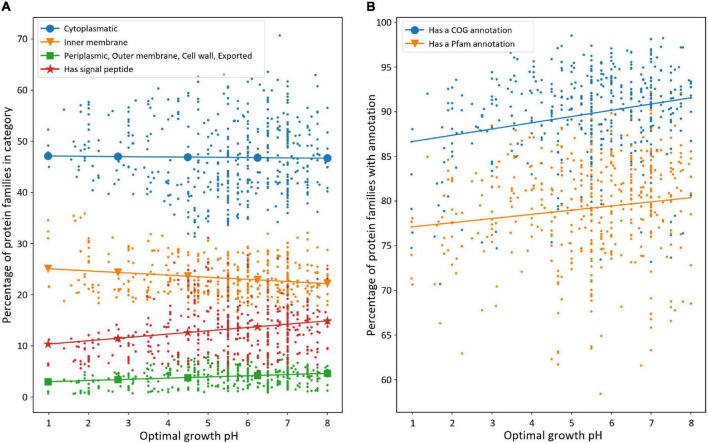
Distribution of protein families across pH. Each point corresponds to a species. **(A)** Subcellular localization and signal peptide presence of protein families across pH. PSORTb and SignalP were used to predict the subcellular location of proteins and signal peptide, respectively. Either subcellular localization or signal peptide presence is expressed in terms of percentage of the protein families (ortholog groups). Pearson’s correlation coefficient and *p*-value, respectively, are −0.01 and 0.77 for cytoplasmic (blue), −0.19 and 4.4 × 10^–5^ for inner membrane (orange), 0.21 and 7.5 × 10^–6^ for periplasmic, outer membrane, cell wall, and exported (green), and 0.22 and 1.4 × 10^–6^ for proteins with a signal peptide (red). **(B)** Percentage of protein families with functional classification across pH. The blue data points and the blue line correspond to proteins with a Clusters of Orthologous Genes (COG) annotation, and the orange data points and the orange line correspond to proteins with a Pfam annotation. Pearson’s correlation coefficients and *p*-values are, respectively, 0.24 and 2 × 10^–7^ for proteins with a COG annotation and 0.14 and 2.6 × 10^–3^ for proteins with a Pfam annotation.

Figure 7A shows the frequency of occurrence of protein families with subcellular location and/or signal peptide predictions expressed as a percentage of the total protein families per genome. The frequency of predicted cytoplasmic proteins does not change across pH. However, there is a statistically significant decrease (Pearson’s correlation coefficient, 0.22; *p*-value, 1.4 × 10^–6^) in the frequency of proteins predicted to have a signal peptide with decreasing pH and a statistically significant increase (Pearson’s correlation coefficient, −0.19; *p*-value, 4.4 × 10^–5^) in the frequency of inner membrane proteins with decreasing pH. There is a small but, nevertheless, statistically significant decrease (Pearson’s correlation coefficient, 0.21; *p*-value, 7.5 × 10^–6^) in the frequency of proteins predicted to be in the category “periplasm, outer membrane, cell wall, and exported” with decreasing pH.

The decrease in proportion of proteins with signal peptides at low pH is consistent with the observation that there are correspondingly fewer proteins predicted in the category “periplasm, outer membrane, cell wall, and exported” at low pH since most of these proteins require a signal peptide export mechanism to pass through the periplasmic membrane (Green and Mecsas, 2016). We hypothesize that the decrease in relative frequency of proteins found outside the inner membrane in acidophiles could be due to physico-chemical challenges that such proteins would encounter as they are exposed to high concentrations of protons at low pH, potentially limiting the diversity of proteins that have evolved to confront such challenges (D’Abusco et al., 2005; Chi et al., 2007; Duarte et al., 2009, 2011; Panja et al., 2020; Chowhan et al., 2021). We speculate that the observed enrichment of predicted inner membrane protein families in acidophiles (Figure 7A) reflects the importance of such proteins in acid stress management since the inner membrane is the barrier that separates the neutral (pH ∼7) cytoplasm from the extreme acid conditions of the periplasm or extracellular space (Slonczewski et al., 2009; Lund et al., 2014; Zhang et al., 2016; Hu et al., 2020; Vergara et al., 2020). This is also supported by the lack of correlation of the representativity of inner membrane proteins with genome size in neutrophiles (Supplementary Figure 5), suggesting that this is a specific adaptation to low-pH environments rather than a general streamlining element.

##### Changes in Ortholog Frequencies by Functional Category

The contribution of gene gain or loss to genome size changes across pH was also analyzed using gene functional classification using COG and Pfam annotations. In total, 25 functional categories are recognized in the 2014 COG database (Galperin et al., 2015), and Pfam v32.0 contains a total of 17,929 families (El-Gebali et al., 2019).^5^ The combination of COG and Pfam analyses provides deep and accurate coverage for searching for predicted protein function in our dataset. Figure 7B shows that the percentage of proteins per genome with a COG or Pfam annotation decreases at a lower pH with statistical significance (Pearson’s correlation coefficients, 0.24 and 0.14; *p*-values, 2 × 10^–7^ and 2.6 × 10^–3^), which is not observed for small neutrophilic genomes (Supplementary Figure 6). This indicates that acidophiles have a higher proportion of putative protein-coding genes that are not recognized by either COG or Pfam. These proteins can be classified as non-conserved, hypothetical proteins with no functional prediction, which do not have protein clusters with sufficient entries to have their own functional annotation in the COG or Pfam databases. It is possible that some of these represent poorly annotated sequences and pseudogenes. However, an intriguing possibility is that some could correspond to validated protein-coding genes that are enriched in acidophiles. Their analysis could potentially yield clues about novel acid tolerance mechanisms and other functions enriched in acidophiles. Examples of such proteins have recently been detected, although their functions remain unknown (González et al., 2016; Vergara et al., 2020).

An analysis of the distribution of functional categories across pH using COGs shows that acidophiles are enriched in several functions that could possibly be attributed to their distinctive metabolisms and environmental challenges (Table 1)—for example, enrichment in proteins assigned to COG L (replication, recombination, and repair) and COG O (chaperone, post-translational modification) might reflect their need for DNA repair and protein refolding when confronted by potentially damaging stresses, such as low pH, high metal concentrations, and oxidative stress (Crossman et al., 2004; Baker-Austin and Dopson, 2007; Cárdenas et al., 2012; Dopson and Holmes, 2014). The increase in the frequency of proteins assigned to COGs C, F, and H (energy production and transport; nucleotide metabolism and transport, and coenzyme metabolism and transport, respectively) could reflect enzyme and pathway requirements associated with obligate autotrophic metabolism that has been found in many acidophiles (Johnson, 1998; Johnson and Hallberg, 2008). As for COG J, it is possible that as ribosomal proteins are very conserved across prokaryotic life (Lecompte et al., 2002), they are less likely to be discarded. Future research could investigate what functions in this category are overrepresented in acidophiles.

**TABLE 1:** Genomic representativity of protein families by function as defined by Clusters of Orthologous Genes (COG) categories in acidophile genomes.

COG category	Pearson’s correlation coefficient	*p*-value
Increased representativity in acidophiles (*p*-value < 0.01)		
(L) Replication, recombination, and repair	−0.25	3.6 × 10^–8^
(F) Nucleotide metabolism and transport	−0.21	5.4 × 10^–6^
(C) Energy production and conversion	−0.21	8.0 × 10^–6^
(H) Coenzyme metabolism and transport	−0.19	3.0 × 10^–5^
(D) Cell cycle control and cell division	−0.16	5.2 × 10^–4^
(J) Translation and ribosome	−0.15	1.1 × 10^–3^
(O) Chaperones, post-translational mod.	−0.13	6.3 × 10^–3^
Decreased representativity in acidophiles (*p*-value < 0.01)		
(S) Function unknown	0.30	1.3 × 10^–10^
(T) Signal transduction mechanisms	0.26	3.4 × 10^–8^

On the contrary, the genomes of acidophiles are depleted in proteins assigned to COG T (Signal transduction mechanisms). A depletion of signal transduction mechanisms has been observed in some marine microbes, especially those that are slow-growing types (Gifford et al., 2013; Cottrell and Kirchman, 2016), in the streamlined genome of the extreme acidophile *Methylacidiphilum infernorum* (Hou et al., 2008) and in the metagenomic profiling data of acidic environments (Chen et al., 2015). The abundancy of signal transduction mechanisms generally declines with decreasing genome size, as it has been found that the number of one- and two-component signal transduction systems is proportional to the square of the genome size (Konstantinidis and Tiedje, 2004; Galperin, 2005; Ulrich et al., 2005). Extensive research has been conducted on the different signal pathways and regulatory networks of acidophiles (Rzhepishevska et al., 2007; Shmaryahu et al., 2009; Moinier et al., 2017; Díaz et al., 2018; Osorio et al., 2019). However, additional research is needed to uncover what signal pathways are not present in these organisms. Acidophiles possess several features which may explain their underrepresentation in proteins from this category, such as having small genomes and having a relatively slow growth speed (Fang et al., 2006; Mykytczuk et al., 2010). The genomes of acidophiles also have a proportionately reduced number of proteins assigned to COG S (unknown function). These are proteins with unknown function that are conserved across multiple species.

#### Paralog Frequency Across pH

We next examined whether the gain or loss of paralogs contributed to genome size changes (mechanism ii, Figure 5A). In contrast to what has been described above concerning gain or loss of specific COG and Pfam gene functions, here we explored how genome size could be influenced by the expansion or contraction of the number of genes in such families. Gene duplication, followed by functional diversification, has been invoked as a major contributor to gene evolution (reviewed in Innan and Kondrashov, 2010; Copley, 2020), and gene paralogs can be present as a significant proportion of a genome (Swan et al., 2013). An increase in the number of paralogous protein copies (including in- and out-paralogs and xenologs; Remm et al., 2001; Darby et al., 2017) has been observed to be correlated with a better performance in a specific function, such as heavy metal resistance or adaptation to other multiple stressors (Kondratyeva et al., 1995; Dulmage et al., 2018). Relatively high paralog frequencies for proteins linked to acid resistance mechanisms have been detected in acidophiles (Ullrich et al., 2016; Vergara et al., 2020).

**FIGURE 8:**
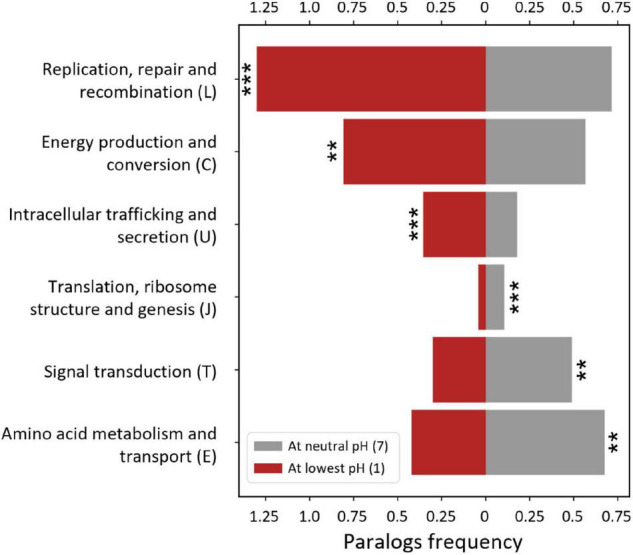
Paralog frequency vs. pH by Clusters of Orthologous Genes (COG) category. The percentage of genes (relative to the proteome size) belonging to paralog families (paralog frequency) was calculated for each COG category. The categories where the paralog frequency had a statistically significant correlation with pH (*p*-value < 0.01) are shown. The mean duplication frequencies at pH 1 and 7 are displayed, calculated with linear regression (Supplementary Figure 7). **p-value < 0.01, ***p-value < 0.001.

We analyzed the paralog frequency changes in genomes across pH by COG categories. The COG annotation has been proved useful for gene enrichment analyses across several genomes (Galperin et al., 2021). As can be seen in Figure 8 and Supplementary Figure 7, acidophiles have relatively high paralog frequencies in the COG categories “replication, repair, and recombination”, “intracellular trafficking and secretion”, and “energy production and conversion” but low frequencies in the COG categories “signal transduction”, “translation and ribosome” and “amino acid metabolism”, as shown by statistically significant correlations (*p*-value < 0.01).

High paralog frequencies were found in the “replication, repair, and recombination” category in acidophiles, which add to their overrepresentation of protein families from this category (Table 1). This might be attributed to a large number of transposases and integrases and also to DNA repair proteins. The high prevalence of mobile elements and horizontal gene transfer in acidophilic genomes has been previously pointed out as key factors for acidophilic evolution (Aliaga et al., 2009; Acuña et al., 2013; Navarro et al., 2013; Ullrich et al., 2016; Zhang et al., 2017; Colman et al., 2018; Vergara et al., 2020). DNA repair proteins have been found to protect against oxidative stress and heavy metal stress, which acidophiles are exposed to in higher levels (Crossman et al., 2004; Baker-Austin and Dopson, 2007; Cárdenas et al., 2012). As for the increased number of paralogous proteins from the “intracellular trafficking and secretion” category, this could result from an abundance of type II secretory systems involved in conjugation or vesicle-related proteins. The former are frequently associated with mobile elements and are particularly abundant in the flexible genomes of acidophiles (Acuña et al., 2013; Beard et al., 2021). In addition, vesicle-related proteins are linked to biofilm formation (Jan, 2017), which, in turn, has been widely observed in acidophiles (Baker-Austin et al., 2010; González et al., 2013; Díaz et al., 2018; Vargas-Straube et al., 2020). Ultimately, a more detailed examination of what specific functions are duplicated is necessary and remains a topic for future research.

Similar to the results of genome representativity (Table 1), the increased paralog frequencies of proteins from the “energy production and conversion” category in acidophiles might be related to their overrepresentation of chemolithotrophic metabolism. Some of the enzymes involved in iron or sulfur oxidation belong to this category, such as cytochrome C, heterodisulfide reductase, and quinone-related proteins (Quatrini et al., 2009; Zhan et al., 2019). Additionally, several proteins in this category are involved in proton exporting functions, such as the H^+^-ATPase, and the overall electron transfer chain proteins, such as ubiquinone oxidoreductase (Walker, 1992; Fütterer et al., 2004; Feng et al., 2015). This indicates that some genes in this category might be in high copy numbers to increase the acid resistance of acidophiles. Alternatively, it could be a consequence of the high energy requirements of maintaining a neutral internal pH (Baker-Austin and Dopson, 2007; Slonczewski et al., 2009).

The reduced paralog frequencies in the “signal transduction” category are concordant with their reduced genome representativity in acidophiles and thus might be accounted by the same phenomena exposed in the previous section about the depletion of these proteins in streamlined organisms. As for the “amino acid transport and metabolism” category, this might be accounted for by a reduction in the number of amino acid importers that are not common in acidophiles. The predominancy of autotrophic metabolism in acidophiles could result in an inclination of these organisms toward the biosynthesis of amino acids rather than uptake by active transporters. Additionally, uptake of amino acids could be harmful to acidophiles as organic acids carry protons into the cytoplasm of these organisms, thus short-circuiting acid resistance mechanisms (Kishimoto et al., 1990; Lehtovirta-Morley et al., 2014; Carere et al., 2021). The current hypothesis is that organic acids are protonated in the extremely acid medium where acidophiles grow (pH < 3), becoming non-ionic and soluble in bacterial membranes and permitting diffusion into the cytoplasm where they uncouple from the proton. A similar phenomenon could occur with amino acids but involve membrane transporters, as amino acids are unlikely to diffuse passively through the membrane.

As for COG J “translation and ribosome,” their reduced paralog frequency is opposite to the increased representativity of protein families from this category in the genomes of acidophiles (Table 1). In other words, acidophiles tend to discard (or not evolve) duplicated genes from this category rather than losing core functions by relinquishing unique protein families. Further exploration is needed to identify the changes that acidophiles exhibit in this category.

Concordantly, as there was an equilibrium between COG categories with increased and decreased paralog frequencies in acidophiles, the overall paralog frequency had no statistically significant correlation with optimal pH and remained at a relatively constant 8% average, ranging from 2 to 20% (Supplementary Figure 8). These relatively low percentages indicate that paralog frequencies are only a minor contributor to genome size changes in our dataset. The constant paralog frequency across pH still contradicts what has been found for other streamlined organisms, which have a relatively low number of paralogs (Giovannoni et al., 2005; Swan et al., 2013). This unusual finding could be partially a consequence of acid resistance genes in multiple copies that would compensate the evolutionary pressure of discarding paralogs.

## Conclusion

We have shown that acidophilic bacteria possess several streamlining features, such as having smaller genomes, fewer ORFs, smaller proteins, and an underrepresentation of signal transduction proteins. Some features that have been described as important in genome reduction in several systems were not detected in acidophiles, such as lower intergenic space percentages and lower overall paralog frequencies. Our study had a statistical approach in contraposition to other streamlining studies which focus on single clades. When considering a dataset of several hundred genomes, our results suggest that the organisms lose genes in the process of adapting to low-pH environments. The reduction in average protein size is an element that has not been the focus of other streamlining studies and is an interesting topic to be developed further in future studies. In addition, several of our findings shed light on the ever-expanding knowledge about acidophile ecology and their acid resistance systems. Mainly, the higher representativity of inner membrane proteins and increased paralog frequencies in COG categories possibly related to energy production, DNA repair, and biofilm formation. The investigation of which functions might be in higher copy number in acidophiles is an interesting topic for future research, as it may uncover novel survival mechanisms for acidophiles. Similarly, acid-related genes shared between acidophiles could be hidden among the proteins without functional annotation.

## Data Availability Statement

The datasets presented in this study can be found in online repositories. The names of the repository/repositories and accession number(s) can be found in the article/Supplementary Material.

## Author Contributions

DC, GN, and DH designed the research and analyzed the data. DC performed the research. DC and DH wrote the manuscript. CG and EV participated in the construction of the final manuscript. All authors read and approved the final manuscript.

## Conflict of Interest

The authors declare that the research was conducted in the absence of any commercial or financial relationships that could be construed as a potential conflict of interest.

## Publisher’s Note

All claims expressed in this article are solely those of the authors and do not necessarily represent those of their affiliated organizations, or those of the publisher, the editors and the reviewers. Any product that may be evaluated in this article, or claim that may be made by its manufacturer, is not guaranteed or endorsed by the publisher.

## Footnotes

1
https://acidb.cl/

2
www.ncbi.nlm.nih.gov

3
img.jgi.doe.gov

4
en.wikipedia.org/wiki/Heteroscedasticity

5
https://pfam.xfam.org
