# Supplementary material for: A Large-Scale Genome-Based Survey of Acidophilic Bacteria Suggests That Genome Streamlining Is an Adaption for Life at Low pH
Source: Front Microbiol. 2022 Mar 21;13:803241. doi: 10.3389/fmicb.2022.803241 (PMC8978632; doi:10.3389/fmicb.2022.803241)
Supplement: Supplementary file 1 [file Data_Sheet_1.PDF]

Supplementary Figures

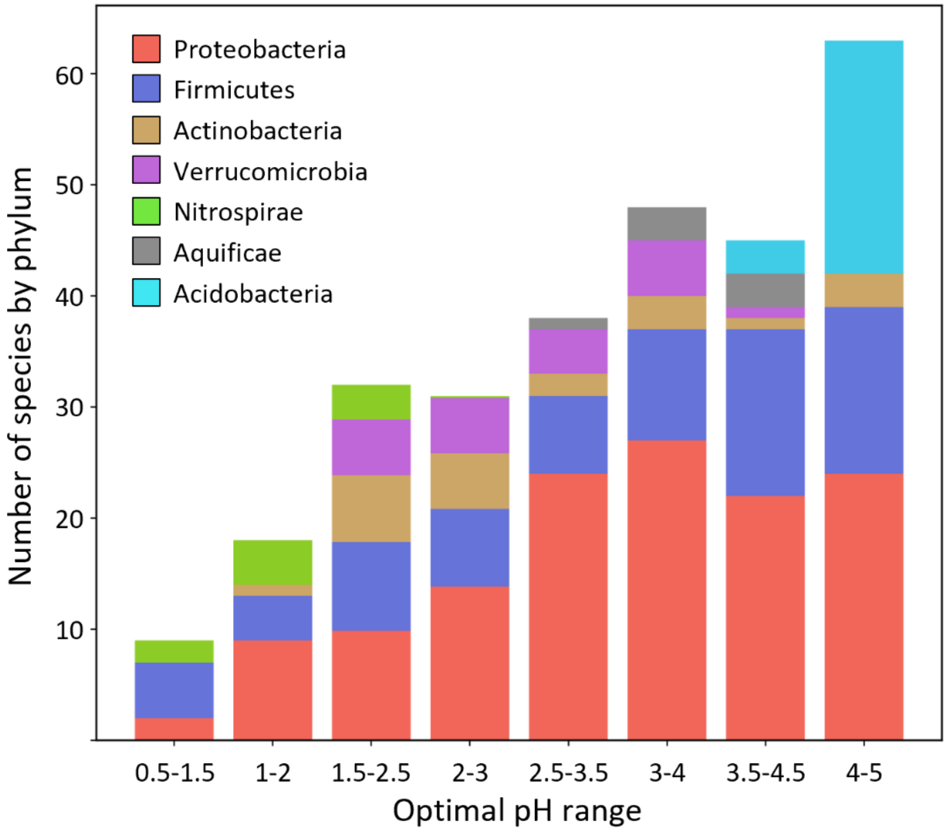

Supplementary Figure 1. Histogram of acidophilic species with sequenced genomes by phylum across pH. Number of species are shown in overlapping increments of one pH unit. Phyla are color coded. Phylum Armatimonadetes has only one acidophilic species and is not shown.

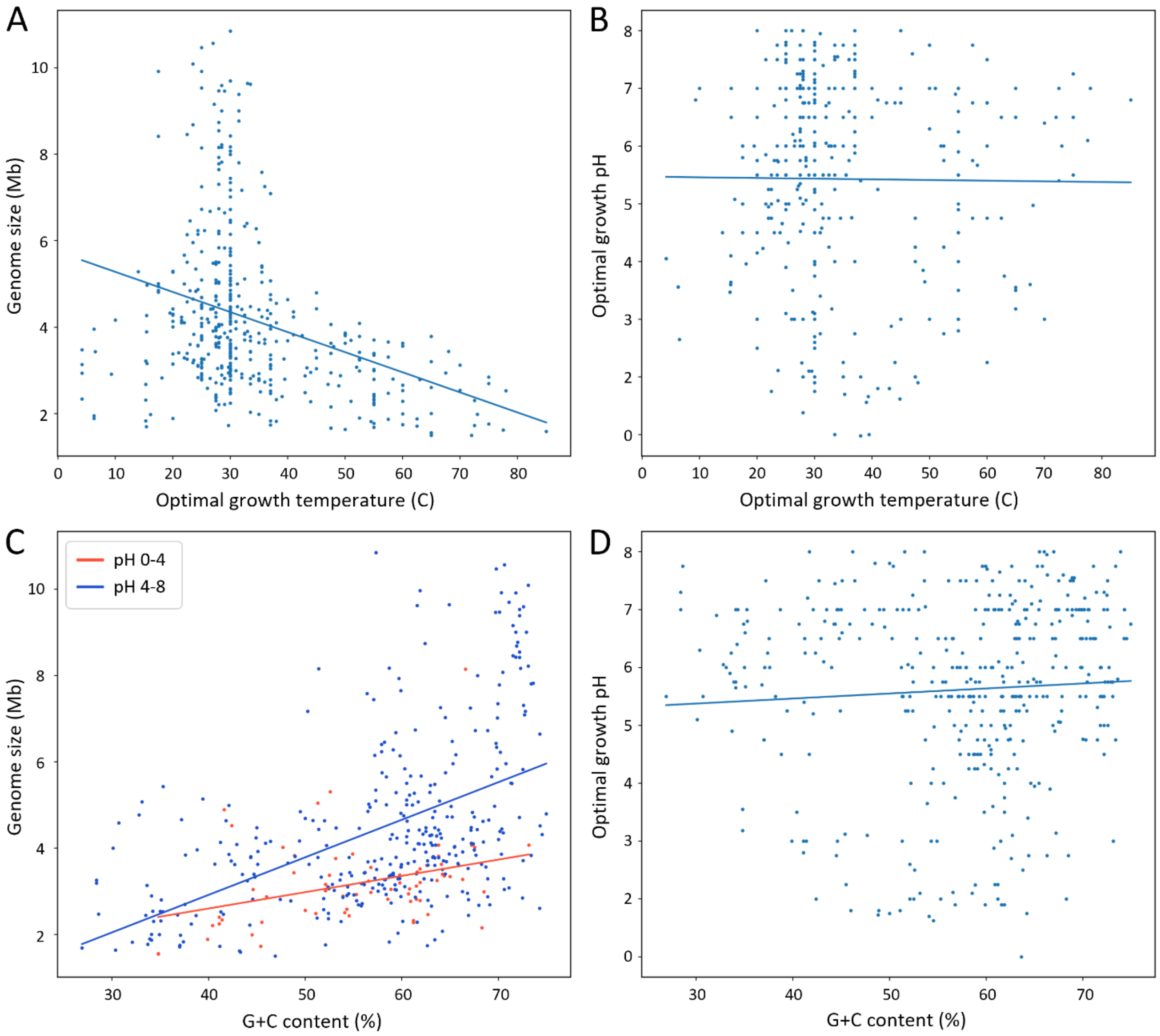

Supplementary Figure 2. Scatterplots showing correlation of genome size and pH versus optimal growth temperature and G+C content of the species in the dataset. (A) Genome size vs optimal growth temperature. Pearson's correlation coefficient is -0.34 with p-value 2.9×10^{-13}. (B) Optimal growth pH versus optimal growth temperature. Pearson's correlation coefficient is -0.01 with p-value 0.84. (C) Genome size versus G+C content. Here, data were separated by pH ranges. Pearson's correlation coefficients were 0.34 and 0.50, with p-values 4.7×10^{-3} and 1.5×10^{-22} respectively for pH 0-4 and pH 4-8. The overall Pearson's correlation coefficient and p-value were 0.48 and 1.91×10^{-25}, respectively. (D) Optimal growth pH versus G+C content. Pearson's correlation coefficient is -0.06 with p-value 0.22.

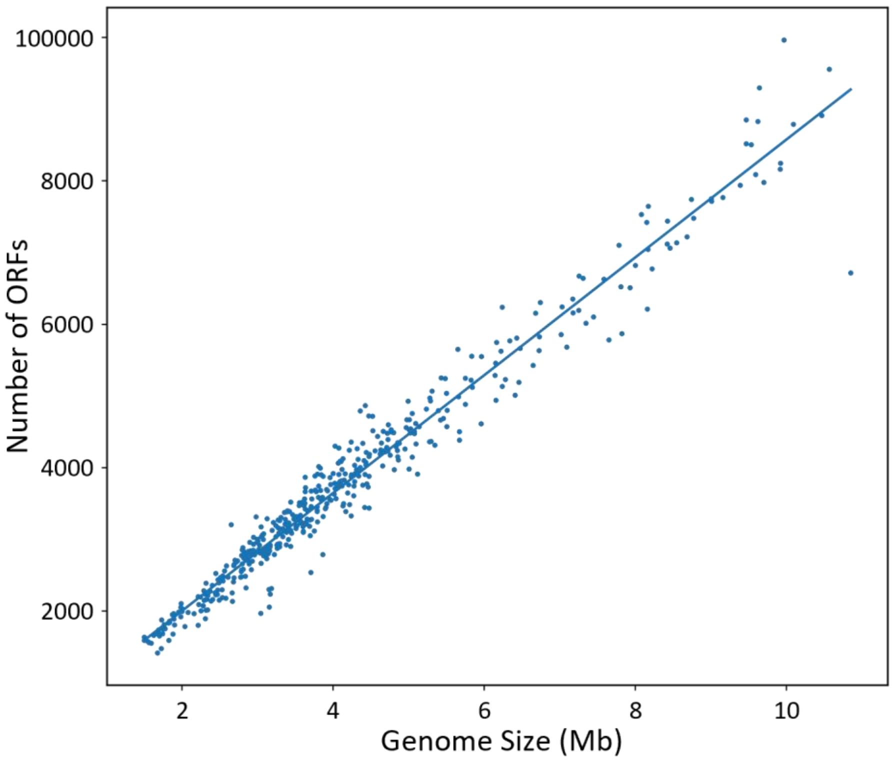

Supplementary Figure 3. Number of ORFs versus genome size. Points are the species averages of the number of ORFs and genome size. Pearson's correlation coefficient is 0.98, with a p-value lower than 10^{-320}.

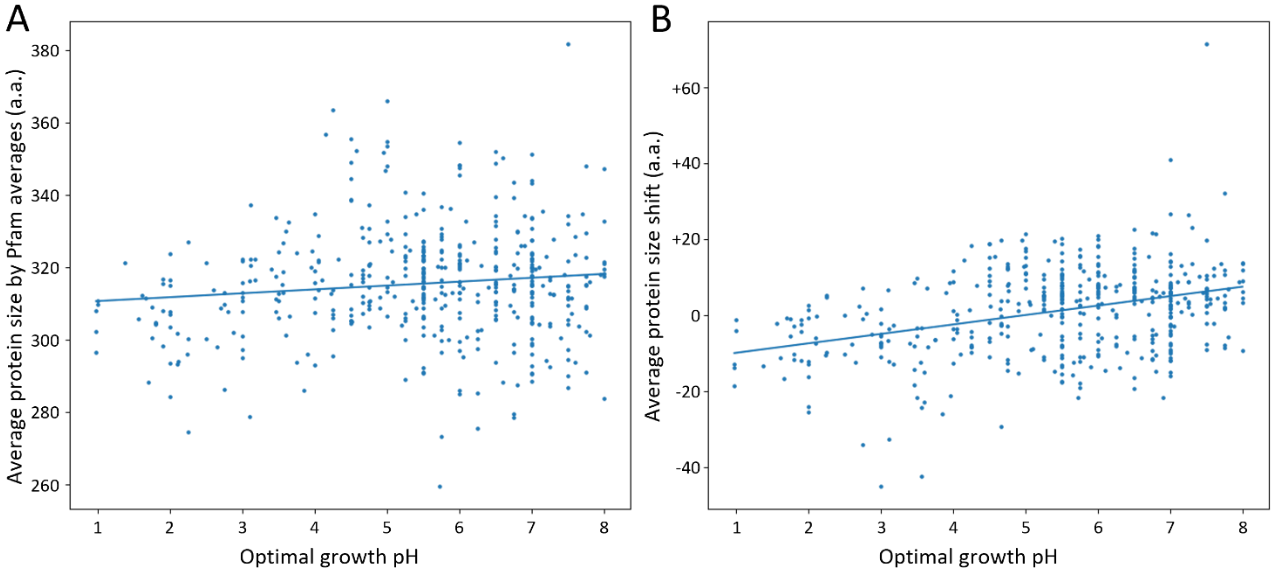

Supplementary Figure 4. Breakdown of the contribution of gene gain/loss and protein size reduction/increase to the observed average protein size changes across pH. The average protein sizes of the different Pfams present in the dataset were first calculated, which was used in both analyses. **(A)** Estimation of the contribution of gene gain/loss. A variation of the average protein size of the proteomes of each species was calculated as follows: for proteins without an assigned Pfam, its protein size was used, while for proteins with an assigned Pfam, the average protein size of their Pfam was used instead. By doing this, all the differences in average protein sizes are due to a different composition in protein families, without the effect of intra-family protein size changes. Pearson's correlation coefficient was 0.12 with a p-value of 0.014, reflecting the contribution of gene gain/loss to the reduction in the average protein size of acidophiles. **(B)** Estimation of the contribution of protein reduction/increase. For the proteomes of each species, the protein size shift in relation to the Pfam averages was calculated for each protein with an assigned Pfam by subtracting the average protein size of its assigned Pfam from its own protein size. For proteins without a Pfam it was 0. The average of all shifts was then calculated. By doing this, all the differences in average protein shift are due to the proteins of an organism being relatively smaller or bigger than other proteins from their same families. Pearson's correlation coefficient was 0.36 with a p-value of 2.54×10^{-15}, reflecting the contribution of protein size reduction to the decrease in the average protein size of acidophiles. "a.a." = amino acids.

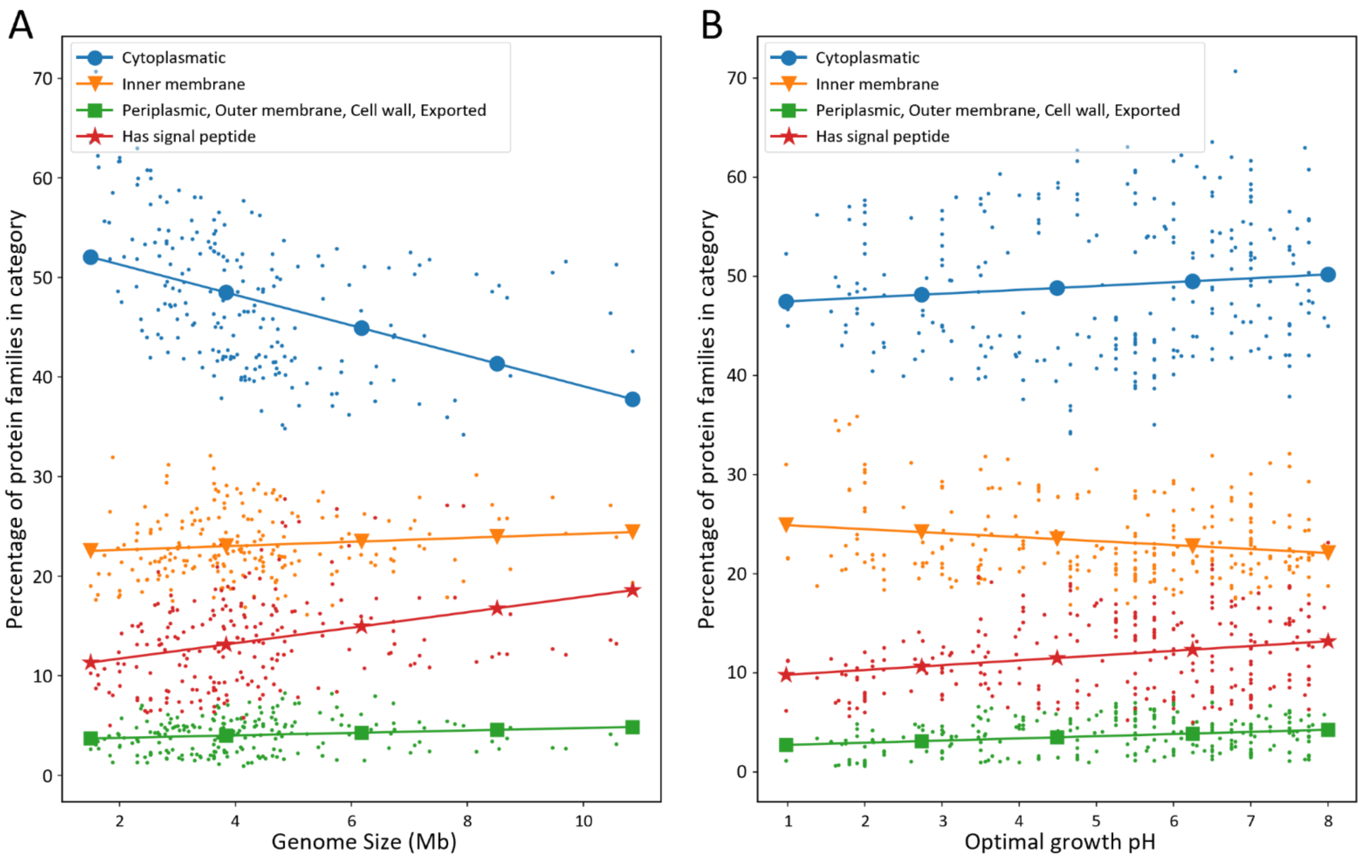

Supplementary Figure 5. Disentangling the effects of genome size changes from subcellular localization correlations. **(A)** Subcellular localization vs Genome size. Analog to Figure 7A but vs genome size and only for neutrophiles (pH 6-8), to isolate the pH influence. Pearson's correlation coefficient and p-value are respectively -0.4 and 2×10^{-9} for cytoplasmic, 0.11 and 0.12 for inner membrane, 0.13 and 0.06 for Periplasmic, Outer membrane, Cell wall and Exported, and 0.3 and 1.3×10^{-5} for proteins with a signal peptide. **(B)** Subcellular localization vs pH in small genomes. Analog to Figure 7A but for genomes under 4 Mb, where there is no correlation between genome size and pH (p-value = 0.15). Pearson's correlation coefficient and p-value are respectively 0.1 and 0.1 for cytoplasmic, -0.19 and 1.8×10^{-3} for inner membrane, 0.24 and 1.3×10^{-4} for Periplasmic, Outer membrane, Cell wall and Exported, and 0.24 and 1.0×10^{-4} for proteins with a signal peptide.

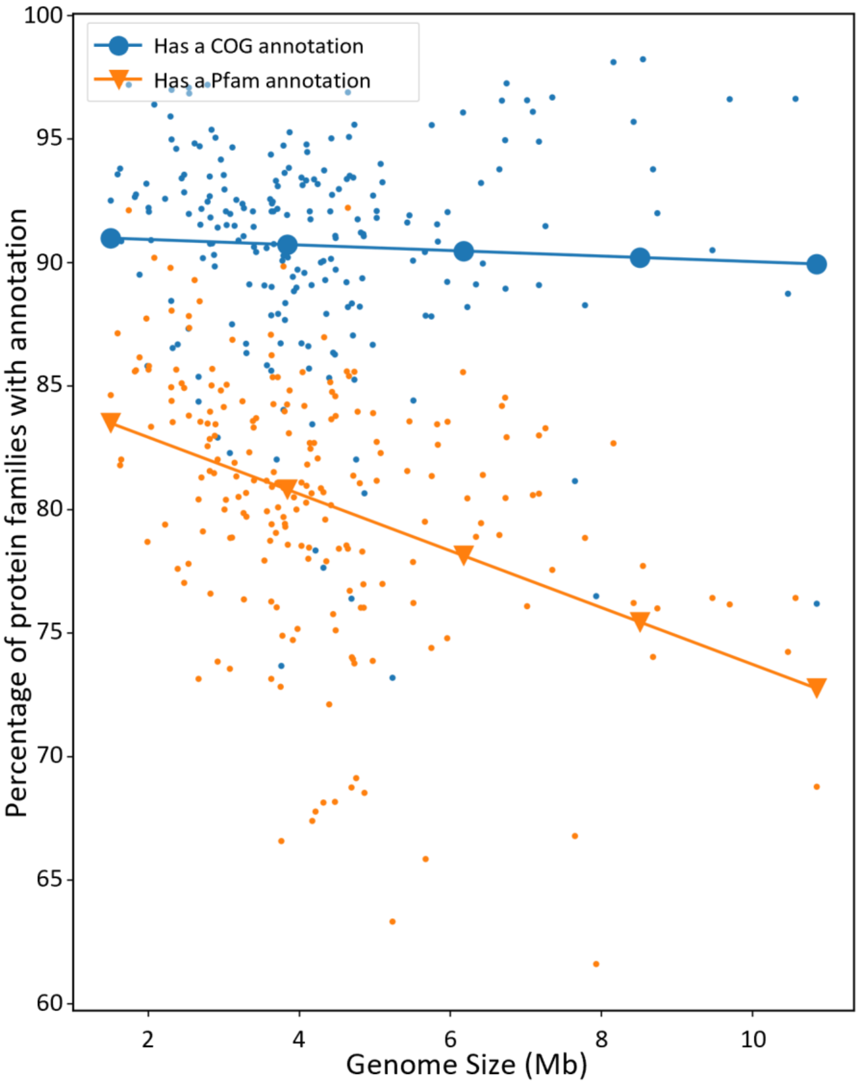

Supplementary Figure 6. Percentage of proteins with a functional annotation versus genome size. The percentage of proteins with either COG annotation or Pfam annotation was plotted against mean genome size. Only neutrophiles (Optimal growth pH 6-8) were considered in this analysis to correct for pH influence. Pearson's correlation coefficients are -0.02 and -0.35 with p-values 0.76 and 2.28×10^{-7} for COG and Pfam annotations, respectively.

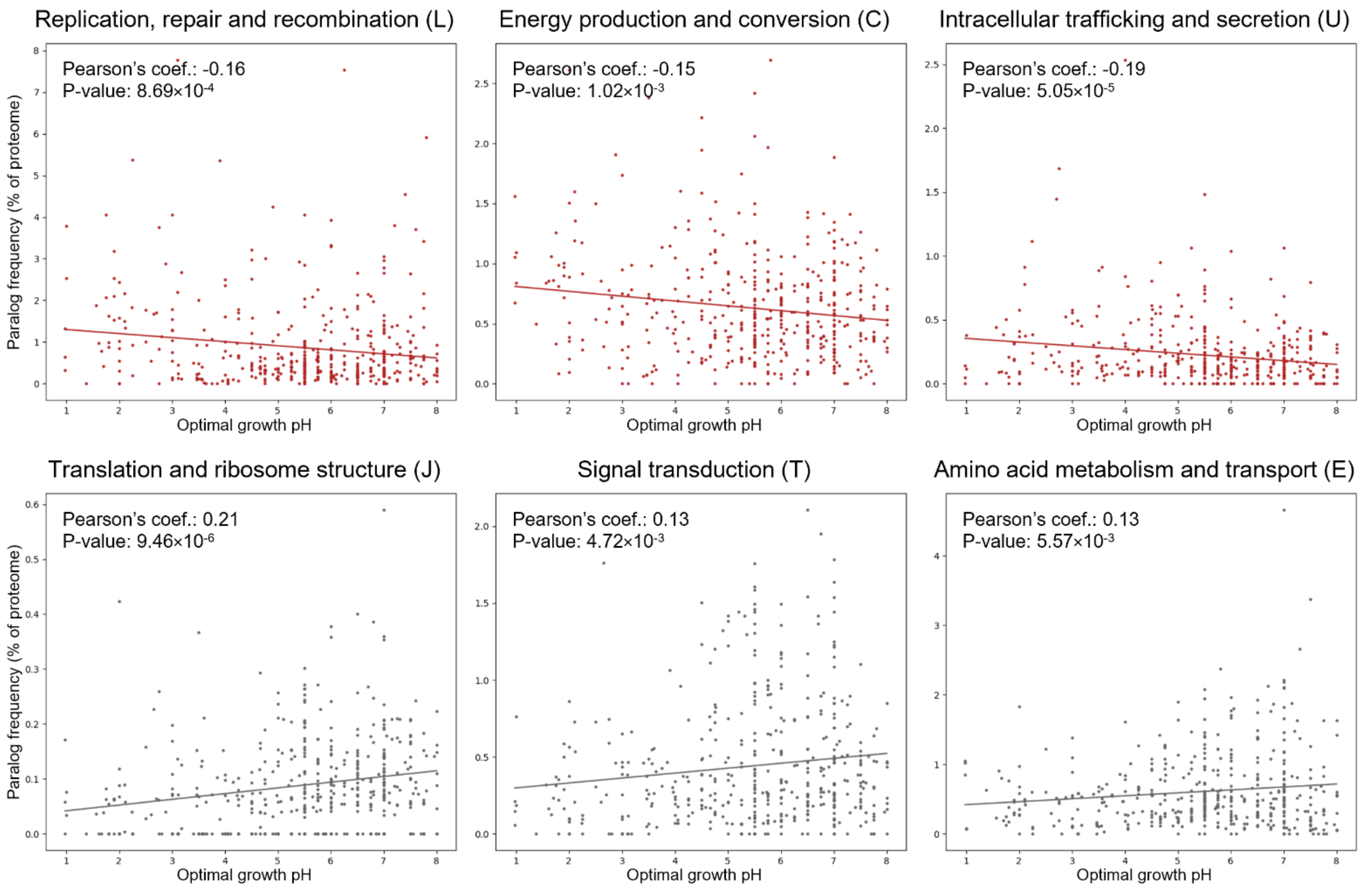

Supplementary Figure 7. Paralog frequencies vs pH by COG category plots. Analog to Figure 8, the individual scatterplot of paralog frequency vs pH for each COG category with statistically significant correlations (p-value <0.01) are shown. Positive correlations are indicated in grey and negative correlations in red. Regression lines are shown, and their projected paralog frequencies at pH 1 and pH 7 were used in Figure 8.

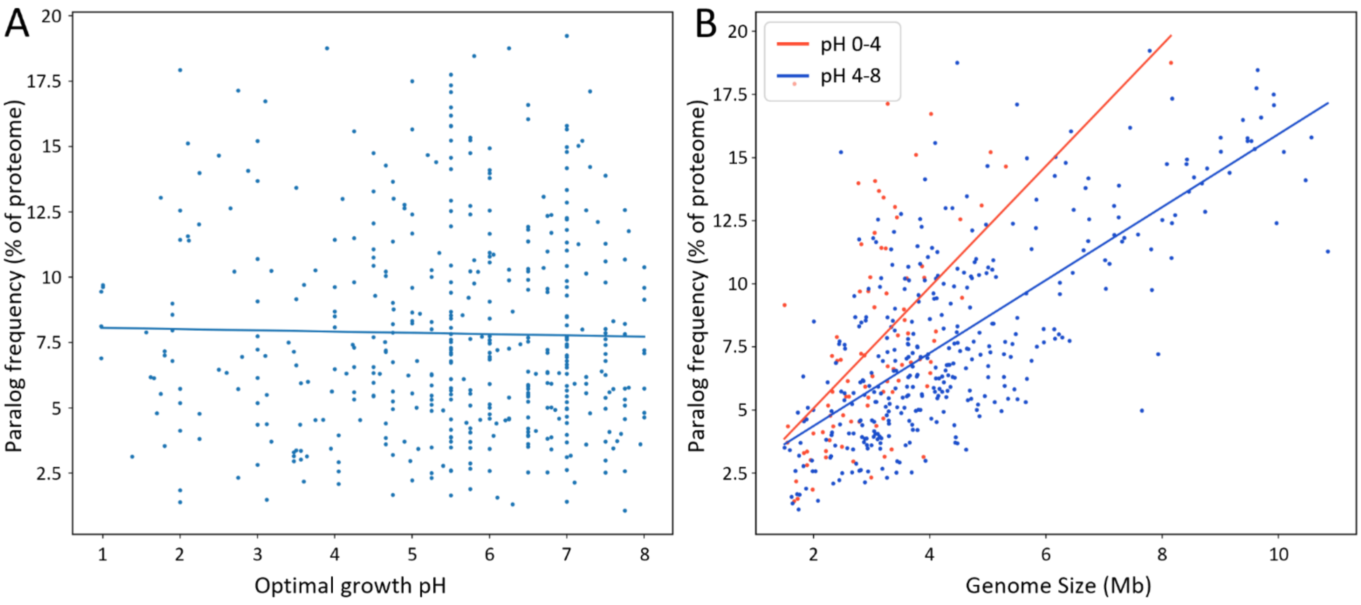

Supplementary Figure 8. General paralog frequency tendencies. **(A)** Paralog frequency vs pH. Ortholog groups with more than one protein in the same genome were defined as paralog groups. The percentage of a proteome that belongs in paralog groups (paralog frequency) was plotted against pH. Pearson's correlation coefficient is -0.02, with p-value 0.67. **(B)** Paralog frequency vs genome size at different pH ranges. Pearson's correlation coefficients and p-values are, respectively: 0.65 and 2.96×10^{-55} for the full range, 0.57 and 4.47×10^{-9} for pH 0-4, and 0.73 and 1.97×10^{-61}.
